# The Deferred Action for Childhood Arrivals program and birth outcomes in California: a quasi-experimental study

**DOI:** 10.1186/s12889-022-13846-x

**Published:** 2022-07-29

**Authors:** Jacqueline M. Torres, Emanuel Alcala, Amber Shaver, Daniel F. Collin, Linda S. Franck, Anu Manchikanti Gomez, Deborah Karasek, Nichole Nidey, Michael Hotard, Rita Hamad, Tania Pacheco-Werner

**Affiliations:** 1 Department of Epidemiology and Biostatistics, UC San Francisco, 550 16th Street, 94143 San Francisco, CA USA; 2 Central Valley Health Policy Institute, California State University, Fresno, Fresno, San Francisco, CA USA; 3 Department of Public Health, UC Merced, Merced, CA USA; 4 Department of Family and Community Medicine, UC San Francisco, San Francisco, CA USA; 5 Preterm Birth Initiative, UC San Francisco, San Francisco, CA USA; 6 Department of Family Health Care Nursing, University of California, San Francisco, CA USA; 7 Sexual Health and Reproductive Equity Program, School of Social Welfare, University of California, Berkeley, Berkeley, CA USA; 8 Department of Obstetrics, Gynecology and Reproductive Sciences, UC San Francisco, San Francisco, CA USA; 9 Cincinnati Children’s Hospital, Cincinnati, OH USA; 10 Immigration Policy Lab, Stanford University, Stanford, CA USA; 11 Philip R. Lee Institute for Health Policy Studies, UC San Francisco, San Francisco, CA USA

**Keywords:** DACA, Birth Outcomes, Quasi-Experimental, Population Health

## Abstract

**Background:**

The Deferred Action for Childhood Arrivals (DACA) program provides temporary relief from deportation and work permits for previously undocumented immigrants who arrived as children. DACA faced direct threats under the Trump administration. There is select evidence of the short-term impacts of DACA on population health, including on birth outcomes, but limited understanding of the long-term impacts.

**Methods:**

We evaluated the association between DACA program and birth outcomes using California birth certificate data (2009–2018) and a difference-in-differences approach to compare post-DACA birth outcomes for likely DACA-eligible mothers to birth outcomes for demographically similar DACA-ineligible mothers. We also separately compared birth outcomes by DACA eligibility status in the first 3 years after DACA passage (2012–2015) and in the subsequent 3 years (2015–2018) - a period characterized by direct threats to the DACA program - as compared to outcomes in the years prior to DACA passage.

**Results:**

In the 7 years after its passage, DACA was associated with a lower risk of small-for-gestational age (− 0.018, 95% CI: − 0.035, − 0.002) and greater birthweight (45.8 g, 95% CI: 11.9, 79.7) for births to Mexican-origin individuals that were billed to Medicaid. Estimates were consistent but of smaller magnitude for other subgroups. Associations between DACA and birth outcomes were attenuated to the null in the period that began with the announcement of the Trump U.S. Presidential campaign (2015-2018), although confidence intervals overlapped with estimates from the immediate post-DACA period.

**Conclusions:**

These findings suggest weak to modest initial benefits of DACA for select birthweight outcomes during the period immediately following DACA passage for Mexican-born individuals whose births were billed to Medicaid; any benefits were subsequently attenuated to the null. The benefits of DACA for population health may not have been sufficient to counteract the impacts of threats to the program's future and heightened immigration enforcement occurring in parallel over time.

**Supplementary Information:**

The online version contains supplementary material available at 10.1186/s12889-022-13846-x.

## Introduction

On June 15, 2012, the Deferred Action for Childhood Arrivals (DACA) program was introduced by an executive branch memorandum [1]. DACA provides temporary protection against deportation and work permits for those who immigrated as children and were undocumented. DACA has undergone substantial legal challenges since its passage [2]. Notably, the Trump administration announced the termination of the program in September 2017 [3]. DACA was upheld by the U.S. Supreme Court in June 2020 on administrative grounds [4] but remains without the protection of Congressional legislation.

Prior research has identified the beneficial impacts of DACA on the health outcomes of recipients [5–7] and their children [8]. One prior study evaluated the short-term impacts of DACA on birth outcomes at a national-level, finding evidence of positive impacts on birthweight outcomes for Mexican-origin mothers in the 2 years following DACA passage [9]. These results may be explained by a number of mechanisms, including the effects of the program on improved employment [10, 11], occupational and educational outcomes [11, 12] and the psychological wellbeing and self-rated health of recipients, including reduced stress related to deportation [5, 6, 13]. In addition, in some states and localities, DACA recipients with qualifying incomes gained expanded access to health care [14], which could have led to improved access to pre-pregnancy and prenatal care for DACA recipients compared to their counterparts who remained undocumented.

Nevertheless, there is evidence suggesting that the population health benefits of DACA may have been attenuated following direct threats to the program under the Trump Presidency and campaign [13, 15]. However, the long-term impacts of DACA on birth outcomes have not been evaluated. In this study, we examined the population-level effects of DACA on birth outcomes using longitudinal data on births in California, the U.S. state with the largest proportion (28.5%) of DACA recipients [16]. We evaluated the impact of DACA on outcomes across the 7 years following DACA passage. However, following a prior study of DACA’s long-term impact on self-rated health [13], we separately evaluated birth outcomes in the immediate 3 years post-DACA passage and the subsequent 3 years. These latter 3 years were characterized by, among other events, the promise of the end to the DACA program during the announcement of the Trump campaign in July 2015 and the announcement of the end to the DACA program in September 2017.

## Methods

### Data

Birth record data spanning 2009–2018 came from the California Department of Public Health’s Birth Statistical Master Files. Analyses were pre-registered at Evidence for Governance and Politics (EGAP) (20190605AB). The Committee for the Protection of Human Subjects, the institutional review board for the California Health and Human Services Agency, and Vital Statistics Advisory Committee approved the study protocol.

### Study sample

We first restricted our data to approximately 3 years before DACA passage through approximately 7 years after DACA passage (June 2009–May 2018), Because there are no direct measures of DACA eligibility or recipient status in California birth records, we followed prior research [6, 17] and used proxy measures of DACA eligibility based on mothers’ birthdate, birthplace, and educational attainment. The DACA memorandum mandated that DACA-eligible individuals were younger than age 31 on June 15, 2012 and had earned a high school degree or GED or were current students, which we used as core criteria to define eligibility. Additionally, DACA eligible individuals must have arrived in the U.S. at age 16 or younger, resided in the U.S. since 2007, and never been convicted of a felony or more than 2 misdemeanors; information on these factors was not available in the birth record.

We restricted the sample to births for which vital statistics data indicated that maternal educational attainment was equal to or greater than high school completion or a GED by the time of delivery and maternal birthplace was one of the top 15 countries of origin for DACA recipients [18]. As of 2017, individuals from these 15 countries of origin accounted for 95.3% of DACA recipients. However, we additionally analyzed outcomes for the subset of births to individuals born in Mexico; Mexican-born individuals comprise 80% of DACA recipients [16, 18] We further restricted our primary analyses to DACA-eligible individuals born within 1 year before vs. 1 year after the DACA birthdate cut-off, which we elaborate on further in our discussion of treatment vs. control groups below.

We restricted the analytical sample to all live singleton births. We excluded birth records with gestational ages < 20 weeks and > 44 weeks and with birthweight for gestational age greater than 3 standard deviations from the sample mean [19]. We excluded 8.6% of observations because of data missing for the following covariates: nativity, date of birth, education, parity, and race/ethnicity of pregnant individuals, and infant sex assigned at birth.

See eFigure 1 for the derivation of the analytic sample.

### Measures

#### Adverse birth outcomes

We evaluated continuous birthweight (in grams), and term birthweight (in grams, among infants born > 37 weeks gestation), and binary outcomes of preterm birth (PTB, < 37 weeks), low birthweight (LBW, < 2500 g), and small-for-gestational-age (SGA) [20]. We used infant sex-specific SGA classifications based on Talge et al. [19]

#### DACA eligibility

We used pregnant individuals’ birthdates to identify likely eligibility for DACA. This improves on the identification strategy of the prior national birth outcomes study that had maternal age rather than maternal birthdate, such that comparison groups were not closely centered around similar birthdates [9]. Specifically, we considered births to individuals born in the year just after the birthdate eligibility cut-off for DACA (i.e. June 15, 1981–June 14, 1982) to be the “treatment” group, and a comparison group of DACA-ineligible individuals born within the year prior to the birthdate cut-off (i.e. June 15, 1980–June 14, 1981) to be the “control” group. Comparing outcomes among these two groups with similar maternal birthdates and otherwise similar demographics helps control for period or age effects.

#### Covariates

We controlled for maternal age and age-squared, a binary indicator of maternal educational attainment (high school graduate or GED equivalent vs. more than high school), infant sex, and parity (1st, 2nd, 3rd, 4th, or > 5th birth). Models also included indicator variables for county, year, and month of birth.

### Statistical analysis

We used a difference-in-differences (DID) design, a quasi-experimental approach well-suited to examining the effects of policies among population subgroups while adjusting for secular trends in a “control” group of similar individuals [21]. In particular, this approach allowed us to “difference out” secular trends among individuals who were otherwise demographically similar (e.g., not U.S.-born, high school graduates) but were ineligible because they were born prior to the DACA birthdate cut-off.

We estimated linear models with robust standard errors in which we regressed each outcome on an indicator of whether the birth was to an individual who was likely DACA-eligible (vs. ineligible) based on their birthdate, an indicator of whether the birthdate fell in the pre-DACA period (June 2009 – May 2012) or the post-DACA period (June 2012 – May 2018), a multiplicative interaction term between these two indicators, and covariates. In order to shed light on potential differences in the long-run impacts of DACA, we alternatively tested a three-category indicator of whether the birthdate fell in the pre-DACA period (June 2009–May 2012), the immediate post-DACA period (June 2012–May 2015) or the period following the start of the Trump U.S. Presidential campaign (June 2015 – May 2018), The primary quantities of interest, which represent the association between DACA and each outcome, are the coefficients for the interaction term between the DACA eligibility and the birthdate timing indicator variables. Linear models for both continuous and binary outcomes are standard for DID analyses because of the different interpretation of interaction terms in non-linear models [22]. Coefficients for binary outcomes can therefore be interpreted as percentage point changes.

We evaluated results among all births regardless of payer type, and then among the subset of respondents with Medicaid as payer. Medi-Cal, California’s Medicaid program, covers prenatal care and labor and delivery for undocumented individuals. We therefore expected that those with Medicaid as payer were more likely than those with other insurance types to include both undocumented individuals eligible for DACA and their counterparts who held similar pre-DACA immigration status but were DACA-ineligible due to the arbitrary birthdate cut-off.

### Robustness checks

Sensitivity analyses (summarized in eTable 1) were designed to evaluate central assumptions of the DID approach: 1) that trends in outcomes for treatment and control groups would otherwise be parallel if it were not for DACA passage and 2) that DACA passage did not contribute to changes in the composition of births for treatment or control groups in the 3 years post-DACA. We also evaluated whether observed associations could plausibly be driven by the one-year difference in average maternal age for DACA eligibility groups. We evaluated year-by-year changes in birth outcomes surrounding DACA passage by switching our binary pre/post-DACA indicator to an indicator of year of birth that spanned 2009–2018 but omitted 2012 given that DACA passage occurred during this year. We carried out the same difference-in-differences procedures as described above with the year of birth indicator. Finally, to shed light on potential mechanisms linking DACA and birth outcomes, we evaluated the association between DACA and prenatal care, using a measure of the number of prenatal visits reported on the birth record as our outcome. We elaborate on the details of these analyses in the Supplemental Appendix.

## Results

### Sample characteristics

In the overall analytic sample, mean age at delivery was about 32 years; DACA-eligible individuals were an average of 1 year younger than their DACA-ineligible counterparts (Table 1). Approximately 55% of births were to individuals with greater than a high school education, 54% of births were billed to Medicaid, and mean parity was 2.3 births (SD: ± 1.1). Male infants accounted for just over half of births.

**Table 1:** Descriptive characteristics by DACA eligibility category, California, June 2009 – May 2018

	DACA Ineligible	DACA Eligible
	Mean (SD) or %	Mean (SD) or %
Maternal age at delivery	32.53 (2.54)	31.59 (2.78)
Maternal education greater than high school	55.4	54.7
Birth billed to Medicaid	53.4	54.9
Parity	2.34 (1.09)	2.29 (1.08)
Male infant	51.0	51.1
Preterm birth	7.3	7.1
Low birth weight	5.0	5.1
Small for gestational age	7.8	8.0
Birth weight, grams	3322 (520)	3323 (521)
Gestational age, weeks	38.61 (1.75)	38.64 (1.73)
Number of prenatal visits	11.94 (3.77)	11.88 (3.69)
Observations	27,898	29,578

Sample includes singleton live-born infants in California with a gestational age of 20 to 44 weeks at delivery, with birthweight for gestational age within three standard deviations of the mean, born to individuals with at least a high school degree and who were born 1-year pre/post the DACA eligibility birthdate cut-off in one of the top 15 DACA-recipient countries

Among infants in the sample, 7% were born preterm, 5% were low birthweight, and 8% were SGA. Mean birthweight was 3323 g (SD: ± 521) for births to DACA-eligible individuals and 3322 g (SD: ± 520) for births to DACA-ineligible individuals. The mean length of gestation was 38.6 weeks (SD: ± 1.7).

### Associations between DACA and birth outcomes

We found some evidence of association between DACA passage and birthweight outcomes in the years post vs. years pre-DACA, although these associations were largely concentrated among Mexican-born mothers and to those whose births billed to Medicaid (Table 2).

**Table 2:** Difference-in-differences estimates of the association between DACA and adverse birth outcomes, California, June 2009–May 2018

	PTB	LBW	SGA	BW	Term BW
	Births to Women from Top 15 DACA Recipient Countries of Origin, All Payor Types				
	β (95% CI)	β (95% CI)	β (95% CI)	β (95% CI)	β (95% CI)
Mother is DACA Eligible*Birth is Post-DACA	0.002	0.000	-0.013*	16.27	18.20
	(−0.009, 0.012)	(− 0.009, 0.009)	(− 0.024, − 0.002)	(−5.30, 37.85)	(−0.323, 36.73)
Observations	57,476	57,476	57,476	57,476	53,351
	Births to Mexican-Born Women, All Payor Types				
	β (95% CI)	β (95% CI)	β (95% CI)	β (95% CI)	β (95% CI)
Mother is DACA Eligible*Birth is Post-DACA	0.005	0.003	−0.009	26.13	33.30**
	(−0.008, 0.018)	(−0.007, 0.014)	(− 0.022, 0.003)	(− 0.08, 52.34)	(10.77, 55.83)
Observations	37,707	37,707	37,707	37,707	35,173
	Births to Women from Top 15 DACA Recipient Countries of Origin, Medicaid Only				
	β (95% CI)	β (95% CI)	β (95% CI)	β (95% CI)	β (95% CI)
Mother is DACA Eligible*Birth is Post-DACA	−0.001	0.002	−0.019*	29.77	29.11*
	(−0.016, 0.013)	(−0.011, 0.014)	(− 0.034, − 0.004)	(−0.14, 59.68)	(3.42, 54.80)
Observations	29,159	29,159	29,159	29,159	27,118
	Births to Mexican-Born Women, Medicaid Only				
	β (95% CI)	β (95% CI)	β (95% CI)	β (95% CI)	β (95% CI)
Mother is DACA Eligible*Birth is Post-DACA	0.000	0.001	−0.018*	45.77**	44.32**
	(−0.016, 0.017)	(−0.015, 0.013)	(− 0.035, − 0.002)	(11.87, 79.68)	(15.20, 73.45)
Observations	22,406	22,406	22,406	22,406	20,887

Notes: Coefficients above represent the interaction between a binary variable for mother’s DACA eligibility and a binary variable indicating the timing of infant birth as pre- vs. post-DACA passage. Covariates include county, year, month fixed effects, maternal age and age-squared, educational attainment, parity, and birth month. *BW* Birthweight, *LBW* Low birthweight, *PTB* Pre-term birth, *SGA* Small for gestational age. * *p* < 0.05, ** *p* < 0.01, *** *p* < 0.001

Among the overall sample, we found evidence of association between DACA and lower risk of small-for-gestational age (β: -0.013, 95% CI: − 0.024, − 0.002). Among births to Mexican-born mothers, we found that DACA was associated with higher term birthweight (β: 33.3 g, 95% CI: 10.77, 55.83) for DACA eligible individuals in the 7 years after the program’s passage.

Among the subset whose births were billed to Medicaid, we found evidence of lower risk of small-for-gestational age (β: -0.019, 95% CI: − 0.034, − 0.004) and greater term birthweight (β: 29.11 g, 95% CI: 3.42, 54.80) for DACA eligible individuals in the years after DACA passage as compared to their non-eligible counterparts.

Among births to Mexican-born mothers whose births were billed to Medicaid, we found evidence of lower risk of small-for-gestational age (β: -0.018, 95% CI: − 0.035, − 0.002) and greater birthweight (β: 45.77 g, 95% CI: 11.87, 79.68) and term birthweight (β: 44.34 g, 95% CI: 15.20, 73.45) for DACA eligible individuals in the years after DACA passage as compared to their non-eligible counterparts.

**Fig. 1:**
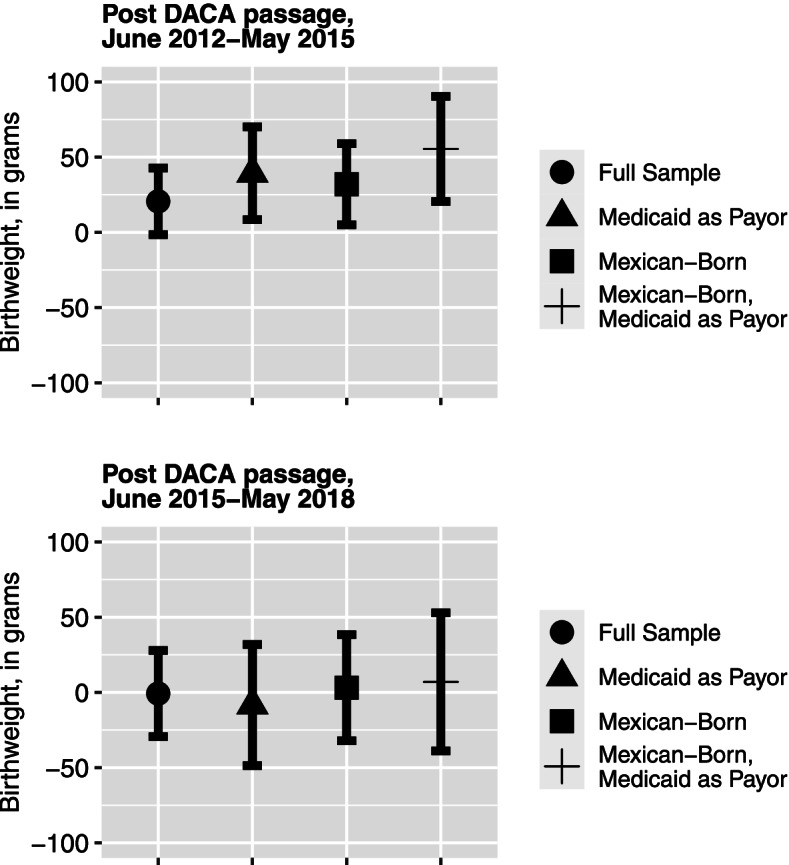
Difference-in-differences estimates of the association between DACA passage and continuous birthweight for likely DACA eligible vs. DACA ineligible individuals in California, 2009–2018

The results of models that instead used a three-category indicator to differentiate between the 3 years immediately following DACA passage and the years following the start of the Trump Presidential campaign (eTable 2, Fig. 1) show estimates of larger magnitude in the 3 years immediately post-DACA passage. There were no differences in birth outcomes for DACA eligible individuals in the period between June 2015 and June 2018 as compared to the pre-DACA period, although confidence intervals were highly overlapping across both the short and long-term periods following DACA passage.

There were no associations observed between DACA and preterm birth, continuous gestational age, or low birthweight for any sub-group at any time point.

### Results of robustness checks

Graphical evaluation supported the DID assumption of parallel trends pre- and post-DACA by mothers’ DACA birthdate eligibility for birthweight outcomes, although the parallel trends assumption appeared to not hold for preterm birth (eFigures 2–6). We found some evidence of association between DACA passage and the composition of births to DACA-eligible vs. ineligible mothers, although this evidence was the weakest for the subset of births covered by Medicaid, for which we observed the largest effect estimates (eTable 2). Specifically, in the overall sample there was evidence that DACA was associated with a lower probability of births being covered by Medicaid. We adjusted for this variable and all other covariates in our models to account for possible confounding.

Associations between DACA and birthweight outcomes for births to Mexican-born mothers and billed to Medicaid were generally robust to “placebo” tests that a) moved the date of DACA implementation 1 year earlier, creating a false policy change date, b) utilized a false maternal birthdate cut-off (i.e., June 19, 1980) to designate mothers’ DACA birthdate eligibility, and c) switched the analytic sample to demographically similar U.S.-born individuals who should not have been impacted by DACA (eTable 4). Results were slightly attenuated but similar when we established a “wash-out” period that excluded births whose gestation spanned the pre- and post-DACA passage periods (eTable 5). We acknowledge, however, that in many cases confidence intervals estimated for placebo tests were overlapping with those estimated in our primary results.

We re-estimated our primary analyses with a year of birth variable in place of the pre/post-DACA indicator (omitting the year 2012) (eTable 6, eFigures 7A-7E). We focused this sensitivity analysis on the subset of births to Mexican-born individuals covered by Medicaid, given that the impacts of DACA appeared most salient for this group in our primary analyses.

While year-by-year estimates were imprecise, these results suggest that there was some divergence in birthweight outcomes in the years post-DACA relative to the reference year of 2009. These differences appeared to be driven by declines in birthweight for those not eligible for DACA rather than by improvements in outcomes for those who were DACA eligible and were most apparent in the years 2014 and 2016, with attenuation of differences in 2017. We also note that, while pre-DACA trends were generally similar across DACA eligibility groups, there was evidence of divergent low birthweight outcomes in 2010 (as compared to 2009) between the two groups.

Finally, analyses of the association between DACA and the number of prenatal visits showed no evidence of differences by DACA eligibility in the post-DACA period (eTable 7). Year-by-year analyses focused on Mexican-origin mothers covered by Medicaid (eTable 6, eFigure 7F) showed some divergence in the average number of prenatal visits, although not in the expected direction: those who were DACA eligible reported fewer prenatal visits than their DACA ineligible counterparts in the years following DACA, with significant differences in 2016 (β: -0.54, 95% CI: − 1.04, − 0.03).

## Discussion

This study provides some of the first evidence of the effect of DACA on birth outcomes and is the first to consider longer-term impacts of the program on birth outcomes during a period of direct threats to the DACA program. Our results suggest that DACA was associated with weak to modest improvements in birthweight outcomes among births to Mexican born individuals and those whose births were billed to Medicaid in the 3 years directly following DACA’s passage compared to 3 years prior. Findings of association between DACA passage and continuous overall and term birthweight were most consistent across analyses, although there was some evidence of association with lower risk of small-for-gestational age for the subset of births billed to Medicaid. These findings are notable, given that even modest differences in infant birthweight have been linked to a wide range of long-term health and developmental outcomes [23–25]. Nevertheless, relatively few significant associations were observed across multiple tests. We also found that the potential benefits of DACA were attenuated in the 3 years marked by the beginning of the Trump campaign and Presidency.

Our findings of stronger associations for the subgroup of DACA-eligible individuals with Medicaid as payer may have been driven by the fact that this group likely had a higher percentage of truly DACA-eligible individuals. This is because California has historically covered both prenatal care and labor and delivery for undocumented individuals under its emergency Medicaid program [26]. Income-eligible DACA recipients were also eligible for full-scope Medicaid, which is comprehensive and could have provided improved access to pre-pregnancy healthcare [14]. While California birth records do not distinguish between emergency Medicaid vs. other Medicaid subtypes, the subgroup with Medicaid as payer may have more closely approximated DACA-eligible individuals who were previously undocumented as well their counterparts who were otherwise similar but would have remained undocumented because they missed the birthdate cut-off for DACA eligibility.

We found no evidence of association between DACA passage and the risk of preterm birth or low birthweight, and the parallel trends assumption appeared to be violated for analyses of preterm birth. The significant short-term associations observed between DACA passage for continuous birthweight outcomes and – in some analyses -- small-for-gestational age for births to individuals using Medicaid could suggest that any potential impacts of DACA may have operated through mechanisms specific to intrauterine growth restriction. Prior studies have suggested that associations between maternal economic and employment circumstances and birth outcomes related to fetal growth could be explained by biological mechanisms of impact on maternal immune and cardiovascular systems as well as on behavioral pathways such as smoking and physical activity during pregnancy [27, 28].

We expected that increased access to care under DACA may be another mechanism of impact on birthweight outcomes. In particular, prior research has uncovered a positive link between Medicaid coverage and low-income women’s maternal and infant outcomes [29, 30]. Immigration policies have also been linked to reductions or improvements in access to care. For example, California’s anti-immigrant Proposition 187 had detrimental impacts on prenatal care utilization, as individuals were afraid to seek healthcare for the fear of deportation [31]. Quasi-experimental research evaluating a policy that expanded healthcare access for pregnant undocumented women was associated with reductions in rates of very low birthweight, but not preterm birth or gestational age [32].

However, the results of our sensitivity analyses ran counter to our hypothesis, suggesting that the average number of prenatal visits following DACA passage were no different for those who were DACA ineligible as compared to those who were DACA eligible. Our year-by-year analyses suggested that in one of the years post-DACA (2016), those who were DACA eligible had *fewer* average prenatal visits compared to their DACA ineligible counterparts. The reasons for this finding are unclear; future research might explore whether these results were driven by fewer pregnancy-related complications for DACA eligible individuals following DACA passage and/or whether these results differ in states without prenatal coverage for undocumented individuals under Medicaid.

Overall, our findings suggest weak to modest impacts of DACA on birthweight outcomes in the short run a specific sub-group. It is important to note that even small impacts on birthweight outcomes can have a wide range of beneficial implications for population health. Nevertheless, there may be multiple reasons for the fact that results were not consistent across birth outcomes and did not persist in the long-term. One potential reason could have been the countervailing adverse impacts of increased immigrant enforcement, including record-level deportations that took place in the U.S. in the years surrounding DACA passage [33]. Research has found significant associations between prenatal exposure to immigration raids and the passage of restrictive immigration policies and birth outcomes [34–37]. The adverse impacts of this restrictive immigration policy context could explain our observation that average birthweight appeared to deteriorate for the DACA ineligible individuals in our sample (eFigures 6D). Moreover, it could be that for many birth outcomes, DACA was not sufficient to fully counteract the impacts of heightened immigration enforcement occurring in parallel.

We additionally found that differences in birthweight outcomes were attenuated to the null in the years following the announcement of the Trump U.S. Presidential campaign in 2015. Year-by-year estimates suggest that results for most outcomes may have been particularly attenuated in the year 2017, following the November 2016 election, although differences in the average number of prenatal visits persisted in 2017. These findings mirror those from a study that found that DACA was linked to improved self-rated health in the 3 years after the program’s passage, but that these benefits eroded after 2015 [13]; another recent study found that significant positive associations between DACA and sleep outcomes attenuated to the null following 2016 [15]. These declining health benefits coincided with uncertainty around the future of the program brought about by the anti-immigration rhetoric and promises to repeal DACA as part of the Trump U.S. Presidential campaign [13]. Nevertheless, confidence intervals surrounding point estimates corresponding to the years immediate after DACA passage and the post-2015 years were substantially overlapping. In addition, year-by-year estimates for some outcomes suggest that by 2018 estimates were similar to those observed in the years immediately following DACA passage. We therefore cannot conclude that birth outcomes were significantly different in the short and long-run after DACA passage.

### Limitations

This study has several limitations, including the use of a proxy method for identifying DACA eligibility, which follows prior studies [6, 8], but could have resulted in misclassification. In particular, the use of a proxy DACA eligibility means that the true percentage of births to individuals who were DACA eligible was smaller than reflected in our analytic sample. This concern is likely most acute for those from countries aside from Mexico. As of 2020, 80% of DACA recipients (over 517,000 enrollees in the U.S.) were born in Mexico [16]. The proportion of DACA recipients from El Salvador, Guatemala, Honduras, Peru, and South Korea is < 5%; the proportion from the remaining top 15 countries is < 1%. This means that the subgroup of individuals born in Mexico may have included a higher proportion of DACA eligible individuals (and DACA recipients) than the broader group of immigrant individuals. Variables on age of arrival and time spent in the U.S. could have helped generate a more precise indicator of eligibility [6, 17]; but are not available in the birth record.

Given the limited precision of our eligibility indicator, we were underpowered to pursue a more robust regression discontinuity design, which would have taken full advantage of the nature of the variation in DACA eligibility [8]. However, we identified this limitation a priori and decided on our alternative difference-in-differences strategy in the pre-registration phase of our study. The difference-in-differences approach has been undertaken by other studies on the population health impacts of DACA in the absence of either sufficient statistical power or a lack of information on respondents’ birthdate needed to carry out a regression discontinuity approach [6, 12].

In addition, our analyses were limited to California. Although California is the U.S. state with the largest proportion of DACA recipients [16], results may not generalize to other states. While California policies were generally supportive of DACA recipients, some states initially blocked DACA recipients from obtaining a driver’s license or paying in-state college tuition [38, 39]. These state-level differences could have led to variation in the degree to which DACA may have led to improvements in population health, including birth outcomes. For example, those in states that chipped away at DACA benefits could have seen fewer positive returns to health; positive health impacts could have been more pronounced in a state like California.

On the other hand, although deportations were at an all-time high in the years surrounding DACA, California had generally refrained from passing anti-immigrant legislation in the years prior to DACA’s passage in contrast to many other states. Protective laws like DACA could have made less of a marginal improvement on population health in a setting already attempting to support immigrants regardless of legal status. A recent national-level study found associations between DACA passage and short-term improvements in birth outcomes [9]. However, the lack of information on specific maternal birthdate in the national data may have led to a somewhat less precise identification strategy. Future research should continue to follow the impacts of DACA on population health at a national level, ideally with data that allows for more precise approximation of DACA eligibility.

Finally, our analyses rest on a set of assumptions that we could not evaluate fully. Policy changes like DACA could have induced shifts in family planning and/or early pregnancy outcomes [40], leading to differences in the composition of births. We tested this assumption with sensitivity analyses of the relationship between DACA passage and maternal demographic characteristics and did find evidence of association between DACA passage and Medicaid coverage. There may have been additional shifts in the composition of births following DACA passage that could not be captured with variables available in the birth record (e.g. driven by pre-conception maternal health or employment, both of which may have been influenced by DACA [6, 10]).

## Conclusion

During a time in which DACA remains without Congressional protection, our study using California data suggests weak to modest short-term impacts of DACA on birthweight outcomes primarily for births to Mexican-born mothers that were billed to Medicaid. Associations with other birth outcomes were null. Any modest positive impacts on birthweight appear to have subsequently been attenuated during a period of heightened anti-immigrant rhetoric and direct threats to the future of the program. These findings have important implications for our understanding of the population health consequences of inclusive immigration policies given that even small improvements in birthweight outcomes may have important downstream consequences for health and development across the lifecourse. Nevertheless, the limited and short-term associations between DACA and birth outcomes identified in our analysis may reflect the fact that any substantial impacts of DACA on population health might have been attenuated by co-occurring restrictive immigration enforcement efforts and/or direct threats to the program’s future.

## Supplementary Information

**Additional file 1.**

## Data Availability

The data that support the findings of this study are available from the California Health and Human Services Agency but restrictions apply to the availability of these data, which were used under license for the current study, and so are not publicly available. Data are however available upon reasonable request from the California Health and Human Services Agency and with permission of the California Health and Human Services Agency.

## References

[1] Napolitano J. Exercising Prosecutorial Discretion with Respect to Individuals Who Came to the United States as Children. 2012. Washington, D.C.: U.S. Department of Homeland Security. https://www.dhs.gov/sites/default/files/publications/s1-exercising-prosecutorial-discretion-individuals-who-came-to-us-as-children.pdf. Accessed 10 Oct 2020.

[2] Duke EC. Memorandum on Rescission Of Deferred Action For Childhood Arrivals (DACA). 2017. Washington, D.C.: U.S. Department of Homeland Security. https://www.dhs.gov/news/2017/09/05/memorandum-rescission-daca. Accessed 10 Oct 2020.

[3] Wolf CF. Reconsideration of the June 15, 2012 Memorandum Entitled “Exercising Prosecutorial Discretion with Respect to Individuals Who Came to the United States as Children”. 2020. Washington, D.C.: U.S. Department of Homeland Security. https://www.dhs.gov/sites/default/files/publications/20_0728_s1_daca-reconsideration-memo.pdf. Accessed 10 Oct 2020.

[4] Department of Homeland Security v. Regents of the University of California, no. 18-587. (U.S. Supreme Court 2020). Accessed 10 Oct 2020.

[5] Patler C, Laster PW. From undocumented to lawfully present: do changes to legal status impact psychological wellbeing among Latino immigrant young adults? Soc Sci Med. 2017;199(C):39-48. 10.1016/j.socscimed.2017.03.009. 10.1016/j.socscimed.2017.03.009 28318760

[6] Venkataramani AS, Shah SJ, O'Brien R, Kawachi I, Tsai AC (2017). Health consequences of the US deferred action for childhood arrivals (DACA) immigration programme: a quasi-experimental study. Lancet Public Health.

[7] Giuntella O, Lonsky J (2020). The effects of DACA on health insurance, access to care, and health outcomes. J Health Econ.

[8] Hainmueller J, Lawrence D, Martén L, et al. Protecting unauthorized immigrant mothers improves their children's mental health. Science. 2017;357(6355):1041-1044. 10.1126/science.aan5893. 10.1126/science.aan5893 PMC5990252 28860206

[9] Hamilton ER, Langer PD, Patler C. DACA's association with birth outcomes among Mexican-origin mothers in the United States. Demography. 2021;58(3):975-985. 10.1215/00703370-9099310. 10.1215/00703370-9099310 34042987

[10] Amuedo-Dorantes C, Antman F (2017). Schooling and labor market effects of temporary authorization: evidence from DACA. J Popul Econ.

[11] Wong T, Richter K, Rodriguez I, Wolgin P. Results from a Nationwide Survey of DACA Recipients Illustrate the Program’s Impact. 2019. National Immigration Law Center, Center for American Progress, United We Dream, and U.S. Immigration Policy Center, UC San Diego. Accessed 10 Oct 2020.

[12] Kuka E, Shenhav N, Shih K. Do human capital decisions respond to the returns to education? Evidence from DACA. Am Econ J: Econ Policy. 2020;12(1):293-324. 10.1257/pol.20180352.

[13] Patler C, Hamilton E, Meagher K, Savinar R. Uncertainty about DACA may undermine its positive impact on health for recipients and their children. Health Aff (Millwood). 2019;38(5):738–45. 10.1377/hlthaff.2018.05495. 10.1377/hlthaff.2018.05495 31059360

[14] Brindis C, Hadler M, Jacobs K, et al. Realizing the dream for Californians eligible for Deferred Action for Childhood Arrivals (DACA): demographics and health coverage. 2014. UC Berkeley Labor Center, UCSF Philip R. Lee Institute for Health Policy Studies, and UCLA Center for Health Policy Research. Accessed 10 Oct 2020.

[15] Giuntella O, Lonsky J, Mazzonna F, Stella L (2021). Immigration policy and immigrants’ sleep. Evidence from DACA. J Econ Behav Organ.

[16] Migration Policy Institute. Deferred Action for Childhood Arrivals (DACA) Data Tools. Accessed October 5, 2020. https://www.migrationpolicy.org/programs/data-hub/deferred-action-childhood-arrivals-daca-profiles

[17] Zong J, Ruiz Soto AG, Batalova J, et al. A profile of current DACA recipients by education, industry, and occupation. 2017. Washington, D.C.: Migration Policy Institute. https://www.migrationpolicy.org/research/profile-current-daca-recipients-education-industry-and-occupation. Accessed 10 Oct 2020.

[18] López G, Manuel-Krogstad J. Key facts about unauthorized immigrants enrolled in DACA. 2017. https://www.pewresearch.org/fact-tank/2017/09/25/key-facts-about-unauthorized-immigrants-enrolled-in-daca/. Accessed Oct 2019.

[19] Talge NM, Mudd LM, Sikorskii A, Basso O (2014). United States birth weight reference corrected for implausible gestational age estimates. Pediatrics.

[20] Battaglia F, Lubchenco L (1967). A practical classification of newborn infants by weight and gestational age. J Pediatr.

[21] Dimick JB, Ryan AM (2014). Methods for evaluating changes in health care policy: the difference-in-differences approach. JAMA.

[22] Karaca - Mandic P, Norton E, Dowd B. (2012). Interaction terms in nonlinear models. Health Serv Res.

[23] Stein RE, Siegel MJ, Bauman LJ (2006). Are children of moderately low birth weight at increased risk for poor health? A new look at an old question. Pediatrics..

[24] Gluckman PD, Hanson MA, Cooper C, Thornburg KL (2008). Effect of in utero and early-life conditions on adult health and disease. N Engl J Med.

[25] McDade TW, Metzger MW, Chyu L, Duncan GJ, Garfield C, Adam EK. Long-term effects of birth weight and breastfeeding duration on inflammation in early adulthood. Proc Biol Sci. 2014;281(1784):20133116. 10.1098/rspb.2013.3116. 10.1098/rspb.2013.3116 PMC4043079 24759854

[26] Kemmick Pintor J, Call KT. State-level immigrant prenatal health care policy and inequities in health insurance among children in mixed-status families. Glob Pediatr Health. 2019;6:2333794X19873535. 10.1177/2333794X19873535. 10.1177/2333794X19873535 PMC6764026 31598542

[27] Eiríksdóttir VH, Ásgeirsdóttir TL, Bjarnadóttir RI, Kaestner R, Cnattingius S, Valdimarsdóttir UA (2013). Low birth weight, small for gestational age and preterm births before and after the economic collapse in Iceland: a population based cohort study. PLoS One.

[28] Dooley D, Prause J. Birth weight and mothers' adverse employment change. J Health Soc Behav. 2005;46(2):141–155. 10.1177/002214650504600202. 10.1177/002214650504600202 16028454

[29] Brown CC, Moore JE, Felix HC, et al. Association of state Medicaid expansion status with low birth weight and preterm birth. JAMA. 2019;321(16):1598–609. 10.1001/jama.2019.3678. 10.1001/jama.2019.3678 PMC6487545 31012935

[30] Montoya-Williams D, Burris H, Fuentes-Afflick E (2019). Perinatal Outcomes in Medicaid Expansion and Nonexpansion States Among Hispanic Women. JAMA.

[31] Spetz J, Baker L, Phibbs C, Pedersen R, Tafoya S (2000). The effect of passing an "anti-immigrant" ballot proposition on the use of prenatal care by foreign-born mothers in California. J Immigr Health.

[32] Swartz JJ, Hainmueller J, Lawrence D, Rodriguez MI (2017). Expanding prenatal care to unauthorized immigrant women and the effects on infant health. Obstet Gynecol.

[33] Gonzalez-Barrera A, Krogstad JM. U.S. Deportations of Immigrants Reach Record High In 2013. 2014. Washington, D.C.: Pew Research Center. https://www.pewresearch.org/fact-tank/2014/10/02/u-s-deportations-of-immigrants-reach-record-high-in-2013/. Accessed 17 Aug 2020.

[34] Novak NL, Geronimus AT, Martinez-Cardoso AM. Change in birth outcomes among infants born to Latina mothers after a major immigration raid. Int J Epidemiol. 2017;46(3):839-849. 10.1093/ije/dyw346. 10.1093/ije/dyw346 PMC5837605 28115577

[35] Torche F, Sirois C. Restrictive immigration law and birth outcomes of immigrant women. Am J Epidemiol. 2018;188(1):24-33. 10.1093/aje/kwy218. 10.1093/aje/kwy218 30358825

[36] Amuedo-Dorantes C, Churchill BF, Song Y. Immigration enforcement and infant health. IZA Discussion Papers, 2020; 13908. Bonn, Germany: IZA - Institute for Labor Economics.

[37] Vu H. I wish I were born in another time: Unintended consequences of immigration enforcement on birth outcomes. Unpublished Manuscript, 2022. https://hoa-vu.github.io/research/HoaVu-JMP.pdf. Accessed 25 May 2022. 10.1002/hec.4775 37910628

[38] National Immigration Law Center. Access to driver’s licenses for immigrant youth granted DACA. 2020. Washington, D.C.: National Immigration Law Center. https://www.nilc.org/wp-content/uploads/2020/06/access-to-DLs-for-immigrant-youth-with-DACA.pdf. Accessed 17 Aug 2020.

[39] National Conference of State Legislatures. Tuition Benefits for Immigrants. 2019. Denver & Washington, D.C. National Conference of State Legislatures. https://www.ncsl.org/Portals/1/Documents/immig/In-State-Tuition-Update-Jan-16-2019.pdf. Accessed 17 Aug 2020.

[40] Kuka E, Shenhav N, Shih K. A reason to wait: The effect of legal status on teen pregnancy. AEA Papers and Proceedings. 2019;109:213-217.

