# Supplementary material for: The Deferred Action for Childhood Arrivals program and birth outcomes in California: a quasi-experimental study
Source: BMC Public Health. 2022 Jul 29;22:1449. doi: 10.1186/s12889-022-13846-x (PMC9338458; doi:10.1186/s12889-022-13846-x)
Supplement: Supplementary file 1 — Additional file 1. [file 12889_2022_13846_MOESM1_ESM.docx]

**Supplemental Appendix for “***The Deferred Action for Childhood Arrivals (DACA) Program and Birth Outcomes in California: A Quasi-Experimental Study*”

| **eTable 1. Summary of Sensitivity Analyses/Robustness Checks** | |
| --- | --- |
| **Sensitivity Analysis** | **Purpose** |
| Visual inspection of DACA trends in annual average birth outcomes for treatment and control groups | Evaluate parallel trends assumption |
| Difference-in-differences estimation of pre vs. post-DACA differences in the *composition* of birth outcomes in DACA-eligible vs. ineligible women | Evaluate the assumption of no compositional differences induced by policy/treatment |
| Difference-in-differences estimation of pre vs. post-DACA differences in birth outcomes, setting birthdate cut-off to be one year prior to true DACA birthdate cut-off | Assess potentially spurious associations driven by differences in average maternal age for treatment and control groups (not pre-registered) |
| Difference-in-differences estimation of pre vs. post-DACA differences in birth outcomes using otherwise same inclusion criteria and DACA birthdate cut-off, but for US-born women | Assess potentially spurious associations driven by differences in average maternal age for treatment and control groups (not pre-registered) |
| Re-estimate difference-in-differences setting DACA passage to one year prior (June 2011) | Evaluate parallel trends assumption (not pre-registered) |
| Re-estimate with a “washout” period, excluding births whose gestation spanned the pre- and post-DACA periods | Reduce potential exposure misclassification |
| Re-estimate difference-in-differences with (instead of a binary pre-post DACA indicator) an indicator of year of birth between 2009-2018, omitting 2012 | Evaluate parallel trends assumption and investigate time-specific trends in post-DACA outcomes. |
| Estimate associations between DACA and the number of prenatal visits (using same difference-in-differences approach) | Evaluate prenatal care as a potential mediator of associations |

**Further description of robustness checks**

To evaluate the assumption of parallel trends in years prior to DACA implementation, we visually inspected trends in birth outcomes in the period prior to DACA implementation (January 2009–June 2012) by plotting the monthly average of birth outcomes (e.g., monthly mean of gestational age) by DACA eligibility. We additionally carried out a placebo test that switched the DACA implementation date to one year earlier; if our results were not driven by pre-trends, we would not expect to see differences in birth outcomes by DACA-eligibility groups using this pre-DACA date.

To evaluate the potential effect of DACA implementation on the composition of births, we conducted the same DID analyses, but set the dependent variables to be each of the maternal or infant characteristics (e.g., maternal education, infant sex). A null result for these analyses would suggest no compositional changes confounding the main results.

Because treatment and control groups had a one-year difference in average age at delivery, which may confound results, we carried out two “placebo” tests to evaluate if any changes in birth outcomes observed in the aftermath of DACA were simply due to differences in age at delivery. First, we switched the birthdate cut-off to be one year prior (June 19, 1980). This generated false “treatment” (June 18, 1980–June 19, 1981) and “control” groups (June 19, 1979–June 19, 1980) that still had an average difference of one year in age at delivery. As neither of the groups in this placebo test would have been eligible for DACA, we would not expect to observe differences in birth outcomes for these groups pre- vs. post-DACA. Second, we evaluated changes in birth outcomes pre- and post-DACA for a sample of US-born individuals (overall and the subgroup of US-born Latinx-identified pregnant individuals) who should not have been affected by DACA, applying the same DACA birthdate cutoffs and otherwise identical inclusion criteria.

| **eTable 2. Difference-in-Differences Estimates of the Association between DACA and Adverse Birth Outcomes, California, June 2009 - May 2018.** | | | | | |  |
| --- | --- | --- | --- | --- | --- | --- |
|  | PTB | LBW | SGA | BW | Term BW |  |
|  | Births to Women from all 15 Top DACA Recipient Countries, All Payer Types | | | | |  |
|  | β (95 % CI) | β (95 % CI) | β (95 % CI) | β (95 % CI) | β (95 % CI) |  |
| DACA Eligible, Birth between June 2012 - May 2015 | 0.001 | -0.002 | -0.012* | 16.929 | 17.947+ |  |
|  | (-0.010, 0.012) | (-0.011, 0.008) | (-0.023, -0.000) | (-4.830, 38.687) | (-0.740, 36.634) |  |
| DACA Eligible, Birth between June 2015 - May 2018 | 0.001 | -0.004 | -0.007 | 2.674 | -0.119 |  |
|  | (-0.012, 0.015) | (-0.015, 0.008) | (-0.021, 0.008) | (-24.665, 30.013) | (-23.726, 23.489) |  |
| Observations | 60122 | 60122 | 60122 | 60122 | 55783 |  |
|  | Births to Mexican-Born Women, All Payer Types | | | | |  |
|  | β (95 % CI) | β (95 % CI) | β (95 % CI) | β (95 % CI) | β (95 % CI) |  |
| DACA Eligible, Birth between June 2012 - May 2015 | 0.004 | 0.001 | -0.008 | 27.069* | 30.349** |  |
|  | (-0.009, 0.017) | (-0.010, 0.012) | (-0.021, 0.005) | (0.521, 53.617) | (7.548, 53.150) |  |
| DACA Eligible, Birth between June 2015 - May 2018 | 0.01 | 0 | -0.005 | 5.926 | 12.337 |  |
|  | (-0.006, 0.027) | (-0.014, 0.013) | (-0.021, 0.012) | (-27.858, 39.710) | (-16.875, 41.550) |  |
| Observations | 39258 | 39258 | 39258 | 39258 | 36597 |  |
|  | Births to Women from all 15 Top DACA Recipient Countries, Medicaid Only | | | | |  |
|  | β (95 % CI) | β (95 % CI) | β (95 % CI) | β (95 % CI) | β (95 % CI) |  |
| DACA Eligible, Birth between June 2012 - May 2015 | -0.004 | -0.005 | -0.018* | 34.660* | 27.210* |  |
|  | (-0.019, 0.011) | (-0.017, 0.008) | (-0.033, -0.003) | (4.302, 65.019) | (1.141, 53.280) |  |
| DACA Eligible, Birth between June 2015 - May 2018 | 0 | -0.003 | -0.011 | -3.322 | -3.844 |  |
|  | (-0.019, 0.019) | (-0.019, 0.013) | (-0.031, 0.008) | (-41.979, 35.336) | (-37.293, 29.605) |  |
| Observations | 30279 | 30279 | 30279 | 30279 | 28137 |  |
|  | Births to Mexican-Born Women, Medicaid Only | | | | |  |
|  | β (95 % CI) | β (95 % CI) | β (95 % CI) | β (95 % CI) | β (95 % CI) |  |
| DACA Eligible, Birth between June 2012 - May 2015 | -0.003 | -0.006 | -0.017* | 50.695** | 41.066** |  |
|  | (-0.020, 0.014) | (-0.020, 0.008) | (-0.034, -0.000) | (16.319, 85.070) | (11.538, 70.594) |  |
| DACA Eligible, Birth between June 2015 - May 2018 | 0.001 | -0.005 | -0.012 | 12.867 | 9.535 |  |
|  | (-0.021, 0.022) | (-0.022, 0.013) | (-0.033, 0.010) | (-31.099, 56.832) | (-28.538, 47.609) |  |
| Observations | 23264 | 23264 | 23264 | 23264 | 21677 |  |
| Notes: Coefficients above represent the interaction between a binary variable for mother’s DACA eligibility and a three-category variable of the timing of infant birth. Covariates include county, year, month fixed effects, mom with > high school education, parity, maternal age and age-squared, mom birth month. BW: birthweight; LBW: low birthweight; PTB: pre-term birth; SGA: small for gestational age. * p<0.05, ** p<0.01, *** p<0.001 | | | | | |  |
|  |  |  |  |  |  |  |

| **eTable 3. Evaluating the association between DACA passage and compositional differences in likely DACA eligible vs. DACA ineligible groups.** | | | | |
| --- | --- | --- | --- | --- |
|  | All Payor Types | | Covered by Medi-Cal Only | |
|  | Model 1: | Model 2: | Model 3: | Model 4: |
|  | All immigrant women | Mexican-born women | All immigrant women | Mexican-born women |
|  | β (95 % CI) | β (95 % CI) | β (95 % CI) | β (95 % CI) |
| Male infant | -0.022* | -0.017 | -0.008 | -0.009 |
|  | (-0.039, -0.004) | (-0.037, 0.004) | (-0.025, 0.009) | (-0.030, 0.011) |
| Parity | 0.013 | 0.009 | 0.022 | 0.010 |
|  | (-0.023, 0.049) | (-0.035, 0.054) | (-0.029, 0.073) | (-0.048, 0.067) |
| Greater than high school education | 0.019* | 0.021* | 0.002 | 0.008 |
|  | (0.002, 0.035) | (0.000, 0.041) | (-0.021, 0.024) | (-0.017, 0.033) |
| Maternal age at delivery | 0.013 | -0.004 | -0.035 | -0.051 |
|  | (-0.036, 0.062) | (-0.063, 0.055) | (-0.102, 0.033)) | (-0.127, 0.025) |
| Medi-Cal | -0.022* | -0.017 |  |  |
|  | (-0.039, -0.004) | (-0.037, 0.004) |  |  |
| Model 1 includes singleton births to mothers from 15 DACA countries, 3 years pre- and 7 years after DACA enactment.  Model 2 restricts analysis to mothers born in Mexico.  Model 3 restricts analysis to births billed to Medi-Cal.  Model 4 restricts analysis to births billed to Medi-Cal and to mothers born in Mexico.  Coefficients above represent the interaction between a binary variable for mother’s DACA eligibility and a binary variable for infant birth after DACA enactment.  BW: birthweight; LBW: low birthweight; PTB: pre-term birth; SGA: small for gestational age  + p<0.10, * p<0.05, ** p<0.01, *** p<0.001 | | | | |

| **eTable 4. Difference-in-Differences Placebo Estimates** | | | | |
| --- | --- | --- | --- | --- |
|  | Placebo 1: | Placebo 2: | Placebo 3: | Placebo 4: |
|  | False Policy Date^a^ | False Birthdate^b^ | US born Covered by Medicaid^b^ | US Born Latina Covered by Medicaid^b^ |
|  | β (95 % CI) | β (95 % CI) | β (95 % CI) | β (95 % CI) |
| PTB | 0.003 | -0.010 | 0.011 | 0.001 |
|  | (-0.016, 0.022) | (-0.028, 0.009) | (-0.001, 0.023) | (-0.017, 0.020) |
| LBW | 0.001 | 0.002 | 0.006 | 0.006 |
|  | (-0.015, 0.017) | (-0.013, 0.017) | (-0.005, 0.017) | (-0.009, 0.021) |
| SGA | -0.011 | -0.003 | 0.002 | -0.003 |
|  | (-0.032, 0.009) | (-0.021, 0.014) | (-0.010, 0.014) | (-0.020, 0.014) |
| BW | 14.737 | -10.566 | -16.050 | -3.282 |
|  | (-26.206, 55.681) | (-47.516, 26.384) | (-40.544, 8.444) | (-39.130, 32.566) |
| Term BW | 15.131 | -20.328 | -6.173 | -0.903 |
|  | (-20.347, 50.610) | (-51.620, 10.963) | (-26.842, 14.496) | (-30.914, 29.109) |
| 1. Sample includes singleton births 3 years before and 3 years after DACA enactment 2. Sample includes singleton births 3 years before and 7 years after DACA enactment   Notes: Model 1 restricts analysis to n = 14971 births (n = 14073 term births) billed to Medicaid and to mothers born in Mexico and sets DACA enactment date 1 year prior to actual DACA passage date (i.e. June 15, 2011).  Model 2 restricts analysis to n = 19741 births (n = 18308 term births) billed to Medicaid and to mothers born in Mexico and sets mother’s eligible birthdate 1 year prior to actual DACA eligibility cutoff.  Model 3 restricts analysis to n = 49837 births (n = 45536 term births) US-born individuals covered by California’s Medicaid program  Model 4 restricts analysis to n = 22994 births (n = 20970 term births) US-born Latinx individuals covered by California’s Medicaid program.  Coefficients above represent the interaction between a binary variable for mother’s DACA eligibility and a binary variable for infant birth after DACA enactment.  Covariates include county, year, month fixed effects, mom with > high school education, parity, maternal age and age-squared, and mother’s birth month.  BW: birthweight; LBW: low birthweight; PTB: pre-term birth; SGA: small for gestational age  *p<0.05, ** p<0.01, *** p<0.001 | | | | |

| **eTable 5. Difference-in-Differences Estimates of the Association between DACA and Adverse Birth Outcomes, California, June 2009 - May 2018, with a Washout Period for Births Born Between June 2012-March 2013; Births to Mexican-born Individuals Covered by Medicaid** | | | | | |  |
| --- | --- | --- | --- | --- | --- | --- |
|  |  |  |  |  |  |  |
|  | β (95 % CI) | β (95 % CI) | β (95 % CI) | β (95 % CI) | β (95 % CI) |  |
| Mother DACA Eligible*Birth is Post-DACA | -0.005 | -0.005 | -0.016 | 48.204* | 41.465** |  |
|  | (-0.023, 0.013) | (-0.020, 0.010) | (-0.034, 0.001) | (11.423, 84.985) | (9.921, 73.010) |  |
| Observations | 20386 | 20386 | 20386 | 20386 | 18996 |  |
| Notes: Coefficients above represent the interaction between a binary variable for mother’s DACA eligibility and a binary variable of the timing of infant birth. Covariates include county, year, month fixed effects, mom with > high school education, parity, maternal age and age-squared, mom birth month. BW: birthweight; LBW: low birthweight; PTB: pre-term birth; SGA: small for gestational age. * p<0.05, ** p<0.01, *** p<0.001 | | | | | |  |
|  |  |  |  |  |  |  |

**eTable 6. Difference in Differences estimates by year, Mexican-born mothers covered by Medicaid**

|  | PTB | LBW | SGA | Birthweight | Term birthweight | Prenatal visits |
| --- | --- | --- | --- | --- | --- | --- |
|  | β (95 % CI) | β (95 % CI) | β (95 % CI) | β (95 % CI) | β (95 % CI) | β (95 % CI) |
| Ref. year = 2009 |  |  |  |  |  |  |
| 2010 | -0.020+ | -0.024* | -0.003 | 28.524 | 8.811 | -0.026 |
|  | (-0.04, 0.00) | (-0.04, -0.00) | (-0.03, 0.02) | (-19.71, 76.76) | (-32.51, 50.13) | (-0.39, 0.34) |
| 2011 | -0.010 | -0.012 | 0.007 | 5.929 | -1.856 | -0.042 |
|  | (-0.03, 0.01) | (-0.03, 0.01) | (-0.02, 0.03) | (-44.04, 55.90) | (-44.71, 40.99) | (-0.42, 0.33) |
| 2013 | -0.012 | -0.015 | -0.009 | 49.377+ | 35.429 | -0.023 |
|  | (-0.04, 0.02) | (-0.04, 0.01) | (-0.04, 0.02) | (-5.73, 104.48) | (-11.91, 82.77) | (-0.44, 0.39) |
| 2014 | -0.017 | -0.036** | -0.021 | 81.981** | 58.950* | -0.426+ |
|  | (-0.05, 0.01) | (-0.06, -0.01) | (-0.05, 0.01) | (23.83, 140.13) | (8.83, 109.07) | (-0.87, 0.02) |
| 2015 | -0.012 | -0.021+ | -0.009 | 30.883 | 15.935 | -0.368 |
|  | (-0.04, 0.02) | (-0.05, 0.00) | (-0.04, 0.02) | (-30.87, 92.63) | (-37.41, 69.28) | (-0.84, 0.10) |
| 2016 | -0.037* | -0.048*** | -0.027 | 91.125** | 30.959 | -0.536* |
|  | (-0.07, -0.00) | (-0.07, -0.02) | (-0.06, 0.01) | (25.50, 156.75) | (-26.01, 87.93) | (-1.04, -0.03) |
| 2017 | 0.015 | 0.006 | 0.008 | -34.826 | -15.340 | -0.450+ |
|  | (-0.02, 0.05) | (-0.02, 0.03) | (-0.03, 0.04) | (-103.96, 34.31) | (-75.20, 44.52) | (-0.98, 0.08) |
| 2018 | -0.022 | -0.040** | -0.020 | 49.491 | 15.696 |  |
|  | (-0.06, 0.01) | (-0.07, -0.01) | (-0.06, 0.02) | (-24.67, 123.65) | (-48.91, 80.30) |  |

Sample includes singleton births and restricts the sample to mothers born 1 year before and 1 year after DACA birthdate eligibility cut-offs.

Covariates include county, year, month fixed effects, mom with > high school education, parity, maternal age and age-squared, and mother’s birth month.

BW: birthweight; LBW: low birthweight; PTB: pre-term birth; SGA: small for gestational age

Note: 2018 data on number of prenatal visits was missing from our dataset

+ p<0.10, * p<0.05, ** p<0.01, *** p<0.001

eTable 7. Difference-in-differences estimates of the association between DACA and the number of prenatal care visits, 2009-2017

|  | Top 15 DACA Recipient Countries of Origin | Mexican-born | Top 15 DACA Recipient Countries of Origin,  Covered by Medicaid | Mexican-born, Covered by Medicaid |
| --- | --- | --- | --- | --- |
|  | β (95 % CI) | β (95 % CI) | β (95 % CI) | β (95 % CI) |
| DACA eligible, births between Jul 2012 – Jun 2015 | -0.109 | -0.101 | -0.123 | -0.166 |
|  | (-0.27, 0.05) | (-0.30, 0.10) | (-0.35, 0.10) | (-0.42, 0.09) |
| DACA eligible, births between Jul 2015 – Jun 2017 | -0.045 | -0.097 | -0.173 | -0.326 |
|  | (-0.26, 0.17) | (-0.36, 0.17) | (-0.48, 0.13) | (-0.68, 0.02) |
| Observations | 54879 | 36181 | 27931 | 21501 |

Sample includes singleton births born in the 3 years before and 7 years after DACA passage and further and restricts the sample to mothers born 1 year before and 1 year after DACA birthdate eligibility cut-offs.

Covariates include county, year, month fixed effects, mom with > high school education, parity, maternal age and age-squared, and mother’s birth month.

BW: birthweight; LBW: low birthweight; PTB: pre-term birth; SGA: small for gestational age

Note: 2018 data on number of prenatal visits was missing from our dataset

* p<0.05, ** p<0.01, *** p<0.001

eFigure 1. Study flow chart

eFigure 2. Visual inspection of the trends in the rate of preterm birth surrounding DACA passage

| 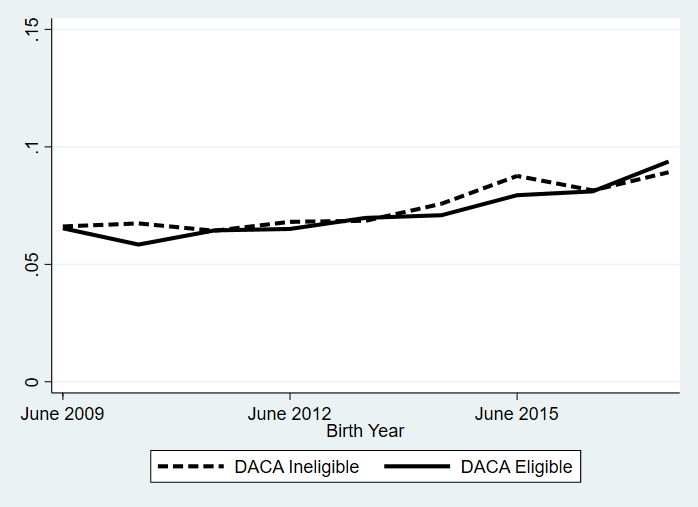 | 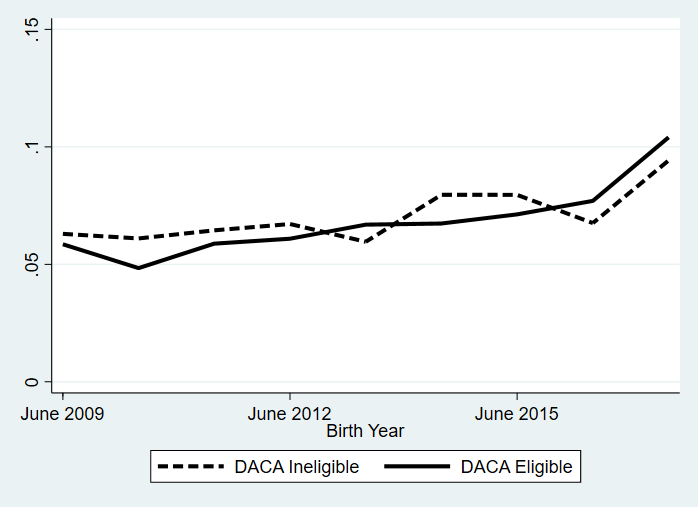 |
| --- | --- |
| Births to individuals from the top 15 DACA recipient countries of origin | Births to Mexican-born individuals |
| 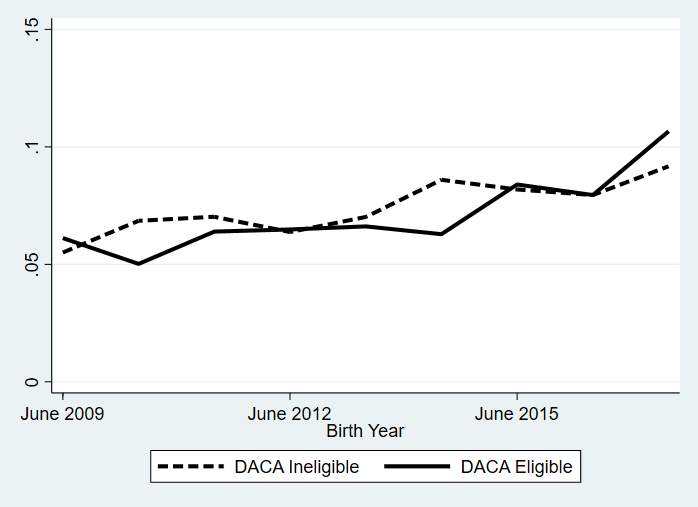 | 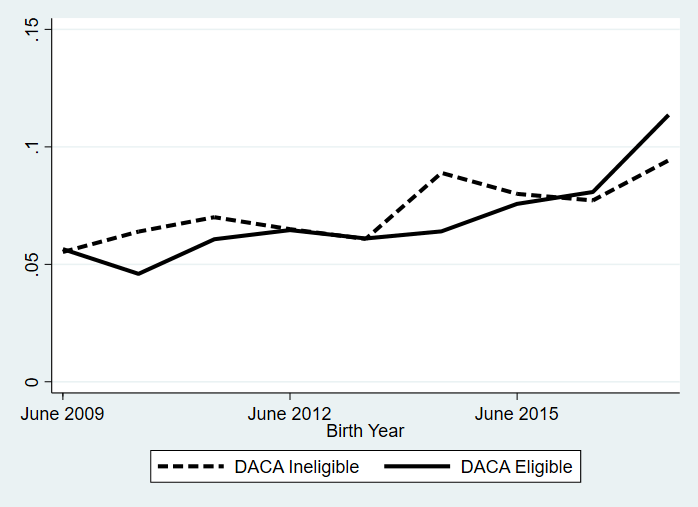 |
| Births to individuals from the top 15 DACA recipient countries of origin, covered by Medicaid | Births to Mexican-born individuals covered by Medicaid |

eFigure 3. Visual inspection of annual trends in the rate of low birthweight surrounding DACA passage

| 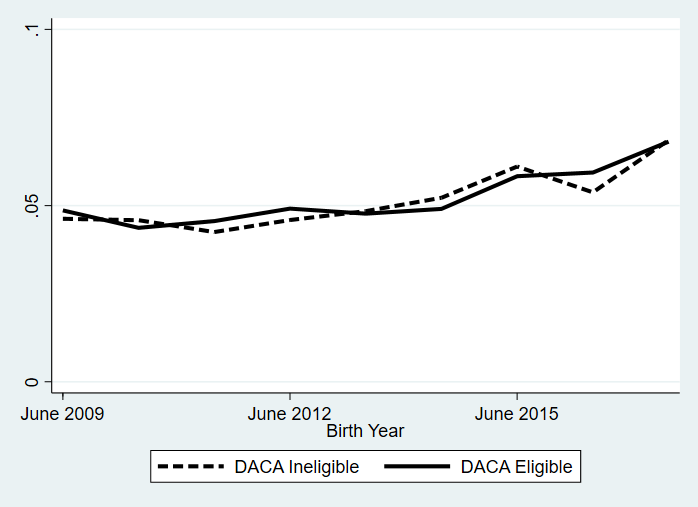 | 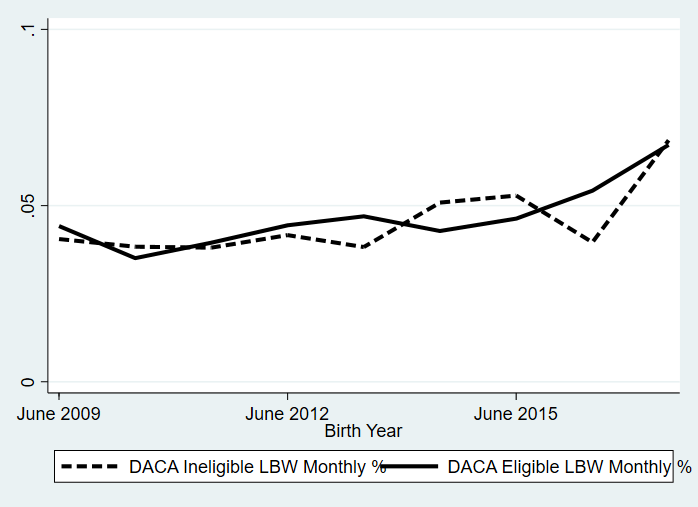 |
| --- | --- |
| Births to individuals from the top 15 DACA recipient countries of origin | Births to Mexican-born individuals |
| 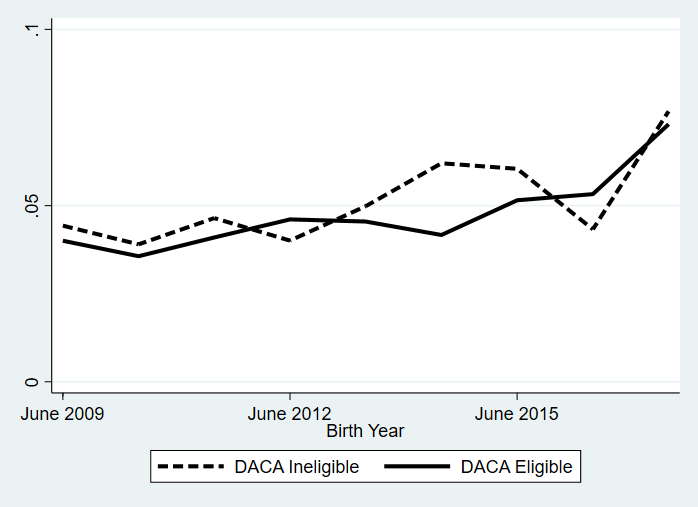 | 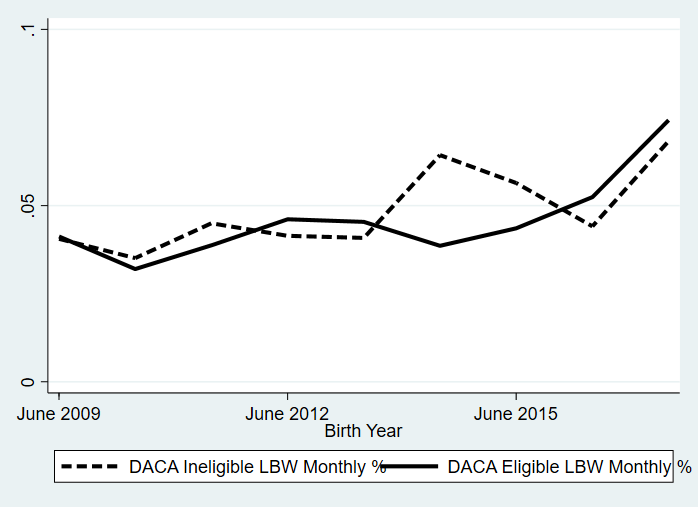 |
| Births to individuals from the top 15 DACA recipient countries of origin, covered by Medicaid | Births to Mexican-born individuals covered by Medicaid |

eFigure 4. Visual inspection of annual trends in average birthweight surrounding DACA passage

| 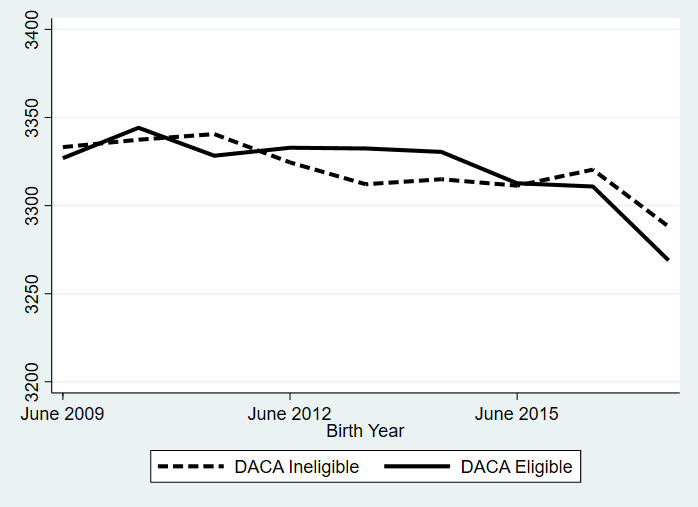 | 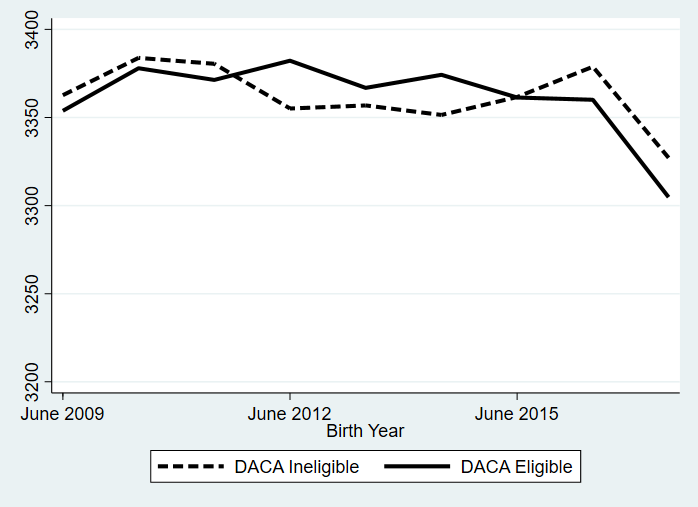 |
| --- | --- |
| Births to individuals from the top 15 DACA recipient countries of origin | Births to Mexican-born individuals |
| 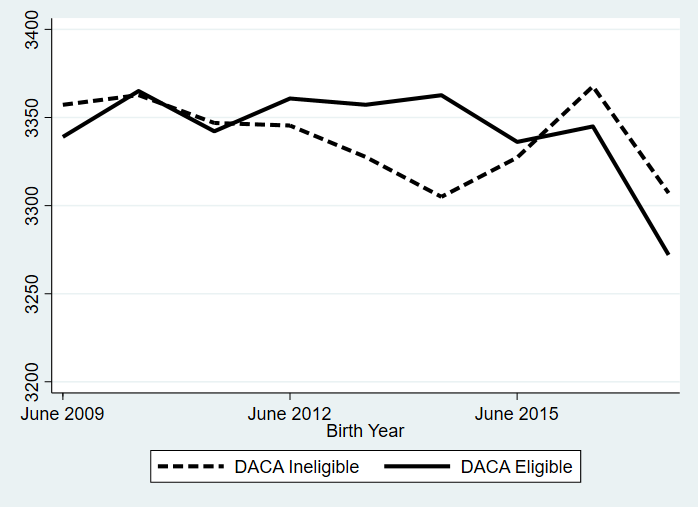 | 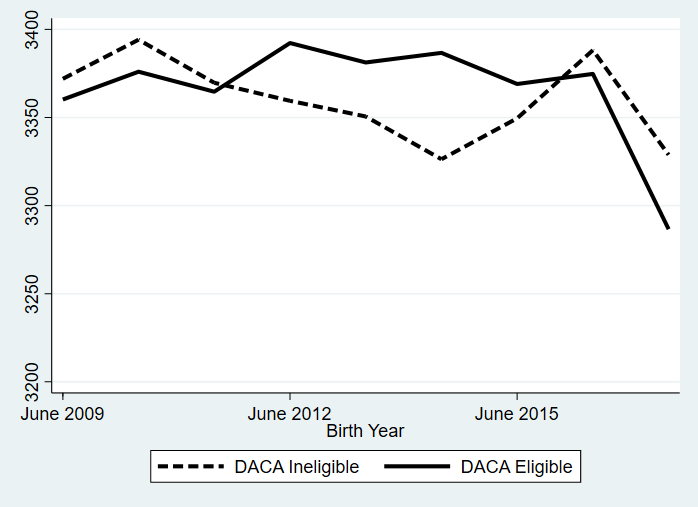 |
| Births to individuals from the top 15 DACA recipient countries of origin, covered by Medicaid | Births to Mexican-born individuals covered by Medicaid |

eFigure 5. Visual inspection of trends in average term birthweight surrounding DACA passage

| 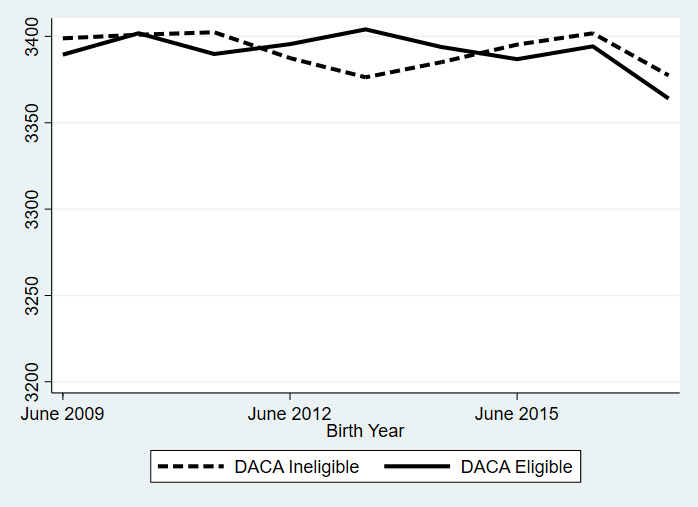 | 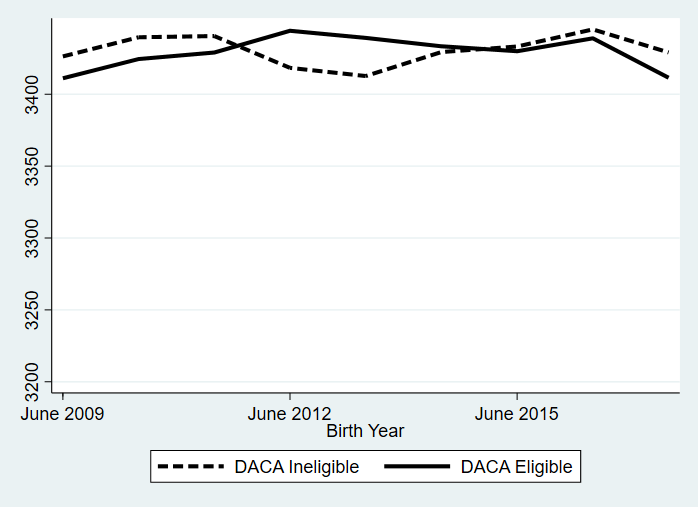 |
| --- | --- |
| Births to individuals from the top 15 DACA recipient countries of origin | Births to Mexican-born individuals |
| 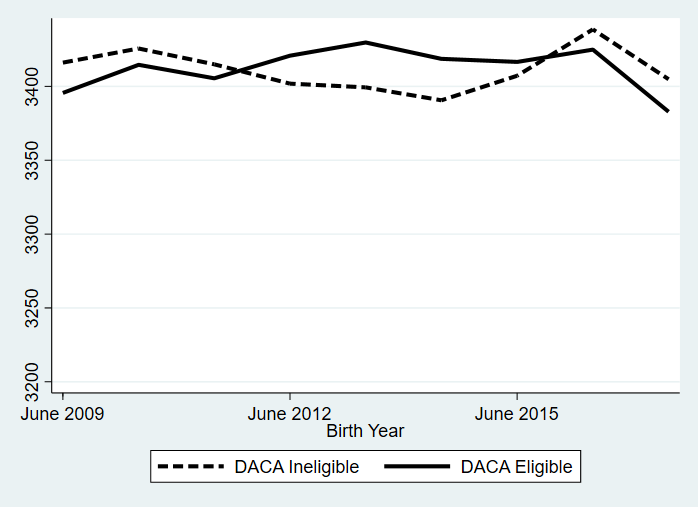 | 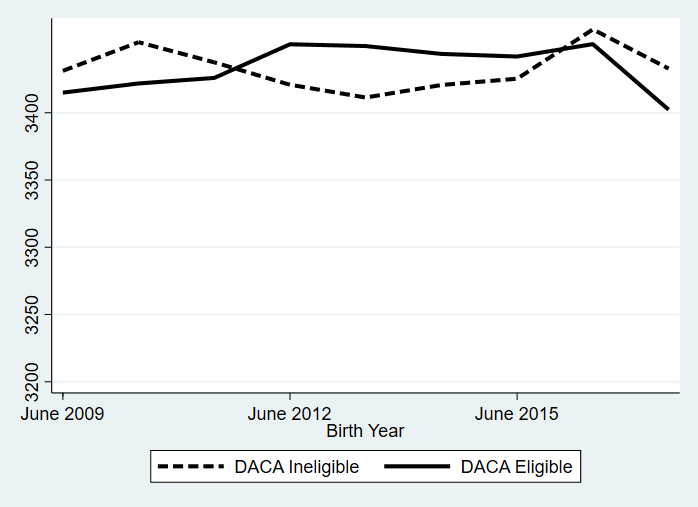 |
| Births to individuals from the top 15 DACA recipient countries of origin, covered by Medicaid | Births to Mexican-born individuals covered by Medicaid |

eFigure 6. Visual inspection of trends in average number of prenatal visits surrounding DACA passage

| 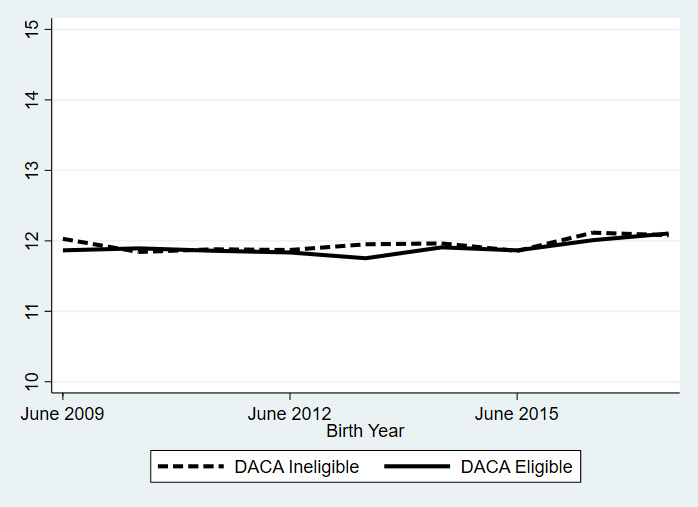 | 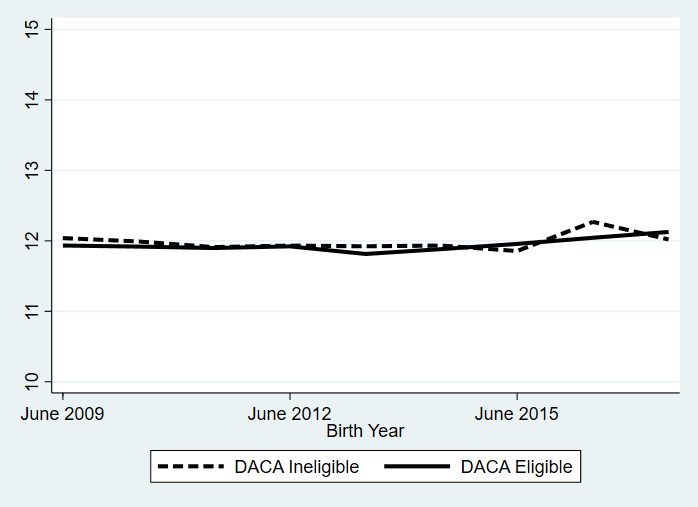 |
| --- | --- |
| Births to individuals from the top 15 DACA recipient countries of origin | Births to Mexican-born individuals |
| 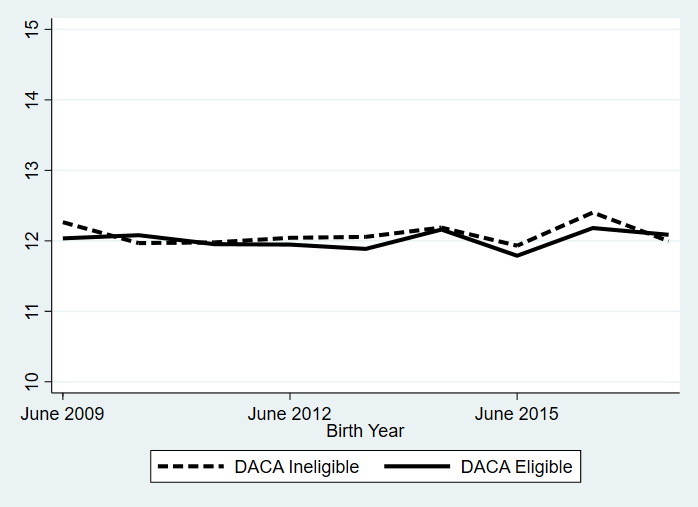 | 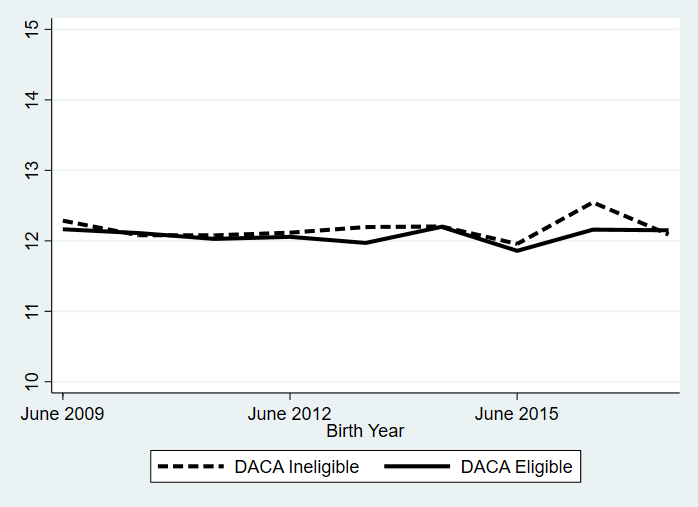 |
| Births to individuals from the top 15 DACA recipient countries of origin, covered by Medicaid | Births to Mexican-born individuals covered by Medicaid |

eFigures 7A-7E. Predicted values of birth outcomes from 2009-2018 by DACA eligibility for Mexican-born mothers covered by Medicaid.

| 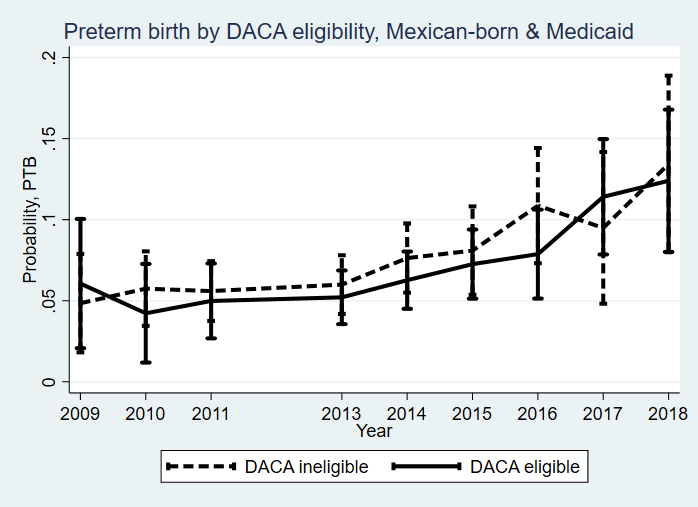  A | 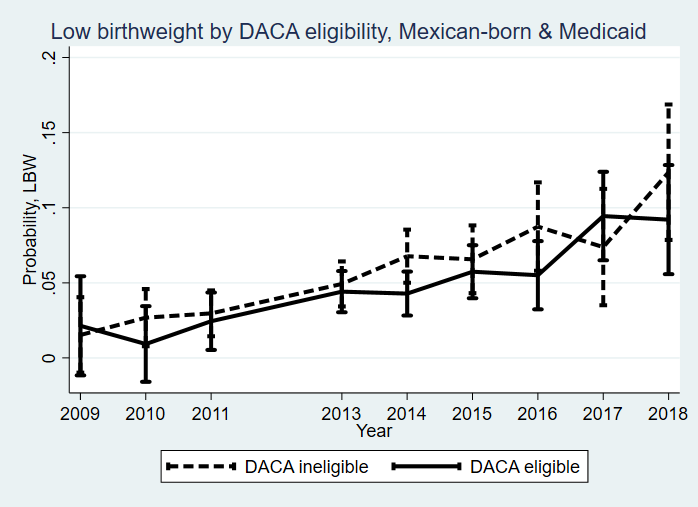  B |
| --- | --- |
| 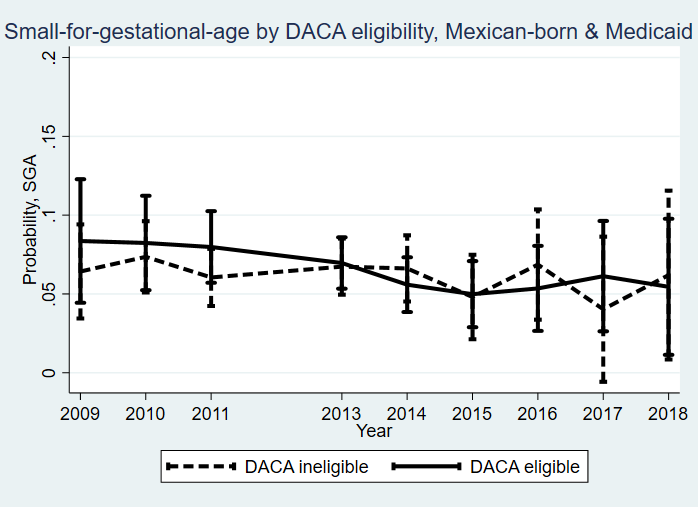  C | 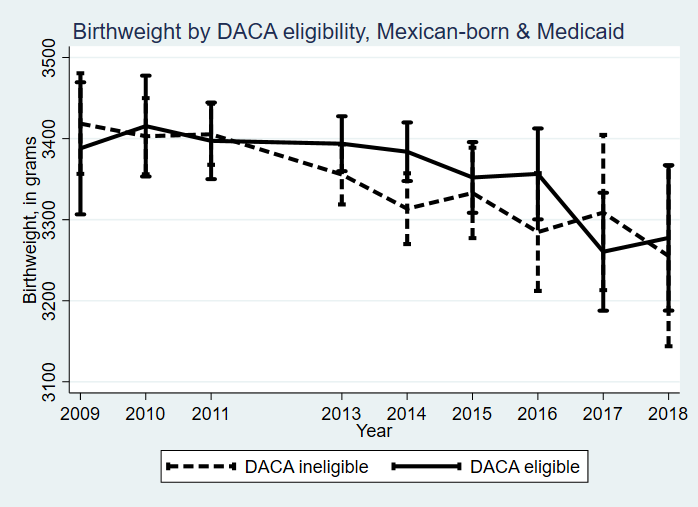  D |
| 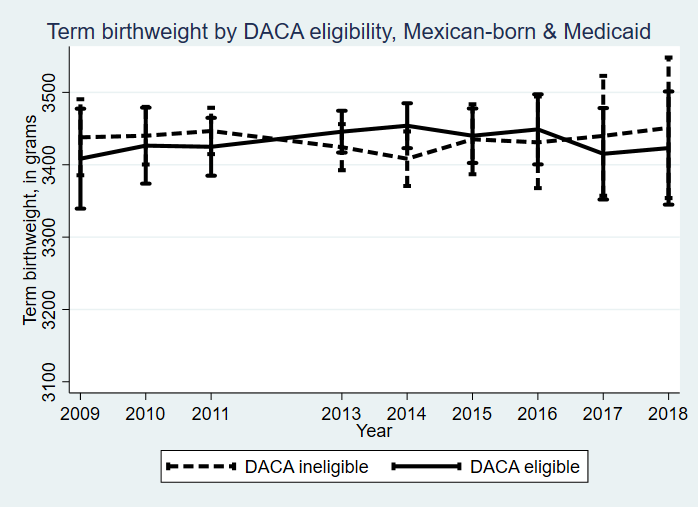  E | 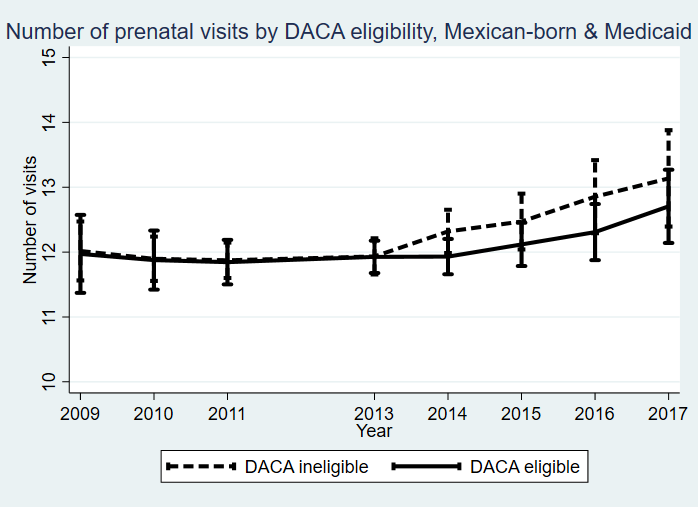  F |
